# ANALYSIS OF ARTHROPLASTIES IN ELDERLY PATIENTS WITH FEMORAL NECK FRACTURES AT A HOSPITAL IN SÃO PAULO

**DOI:** 10.1590/1413-785220263403e299827

**Published:** 2026-06-12

**Authors:** Marcos Gabriel Falcão, Marcelo Añez Suarez, Anna Clara Oliveira Paranhos Ferreira, Lucas Verissimo Ranzoni, Tajher Iunes, Alberto Maranon Terrível

**Affiliations:** 1 Hospital IGESP, Sao Paulo, SP, Brazil.

**Keywords:** Hip Arthroplasty, Fractures, Bone, Femur, Postoperative Care, Postoperative Complications, Artroplastia De Quadril, Fraturas Ósseas, Fêmur, Cuidados Pós-Operatórios, Complicações Pós-Operatórias

## Abstract

**Objective:**

To analyze the outcomes related to partial and total hip arthroplasties in elderly patients with femoral neck fractures at a hospital in the city of São Paulo.

**Material and methods:**

We reviewed the medical records of patients undergoing hip arthroplasty between 2020 and 2024 and extracted data on demographics, type of arthroplasty, length of hospital stay, and any postoperative complications such as infection and death.

**Results:**

198 patients, mean age 84 years. Women (80.2%), men (19.8%). The mean time to surgery was 2.3 days, with 71.6% undergoing surgery within two days (early surgery). Length of ICU stay was significantly longer (p = 0.002) in the late surgery group (M = 47, SD = 40) compared to the early surgery group (M = 31, SD = 41). The total length of hospital stay was longer in the Late Surgery group (M = 17 days) compared to the Early Surgery group (M = 7 days), with statistical significance (p = 0.001) and a moderate-to-large effect size (ES = 0.695).

**Conclusion:**

Late surgery in elderly patients with femoral neck fractures increases hospitalization and ICU length of stay, but does not affect complications, mortality, or functional recovery. Although early surgery reduces hospitalization, management requires a multifactorial approach. ***Level of Evidence IV; Retrospective Cohort.***

## RESUMO

**Objetivo:**

Analisar os desfechos relacionados a artroplastias parciais e totais em idosos com fratura do colo do fêmur em serviço hospitalar da cidade de São Paulo.

**Métodos:**

Realizamos revisão de prontuários dos pacientes submetidos a artroplastia de quadril no período entre 2020 e 2024 e extraímos dados demográficos, tipo de artroplastia, tempo de internação e eventuais complicações pós-operatórias como infecção e óbitos.

**Resultados:**

198 pacientes, média de 84 anos. Mulheres (80,2%), homens (19,8%). O tempo médio até cirurgia foi 2,3 dias, com 71,6% operados em até dois dias (cirurgia precoce). Tempo de permanência em UTI foi significativamente maior (p = 0,002) no grupo de cirurgia tardia (M = 47, DP = 40) em comparação ao grupo precoce (M = 31, DP = 41). O tempo total de internação foi maior no grupo Cirurgia Tardia (M = 17 dias) em comparação ao grupo Precoce (M = 7 dias), com significância estatística (p = 0,001) e tamanho de efeito moderado-alto (TE = 0,695).

**Conclusão:**

A cirurgia tardia em idosos com fratura do colo do fêmur aumenta o tempo de internação e UTI, mas não afeta complicações, mortalidade ou recuperação funcional. Embora a cirurgia precoce reduza a hospitalização, o manejo exige abordagem multifatorial. ***Nível de Evidência IV; Coorte Retrospectiva.***

## INTRODUCTION

Femoral neck fractures are a public health problem, with a global incidence estimated at 1.6 million cases annually, projected to reach 4.5 million by 2050 due to population aging.^1^ In Brazil, data from DATASUS reveal approximately 589,826 hospitalizations from 2019 to 2023, ranging from 109,189 admissions in 2019 to 131,425 in 2023, with a constant increase during this period.^2^ It is a fracture with a bimodal peak incidence, occurring in young individuals due to high-energy trauma, such as car accidents, or in low-energy trauma, predominantly in elderly individuals from falls from their own height.^3^

These fractures are a rupture of the cortical bone between the femoral head and the greater trochanter, classified by Garden according to the degree of displacement and alignment of the bone trabeculae.^4^ The diagnosis is usually made with X-rays in anteroposterior and lateral views of the hip, with high sensitivity. However, in cases with clinical findings that diverge from imaging results, such as in the suspicion of occult fractures, the literature indicates the use of Magnetic Resonance Imaging (MRI).^5^

Surgical treatment is the gold standard, with two main surgical modalities: osteosynthesis and arthroplasty. The surgical definition depends on several factors, such as the fracture pattern, degree of prior functionality, life expectancy, and surgical risk. For osteosynthesis, one option is cannulated screws, which are indicated for non-displaced fractures, preserving the bone anatomy. Total or partial arthroplasty is indicated for unstable and displaced fractures, but it comes with a higher financial cost related to the implant.^6–9^

Surgical treatment for these fractures is based on achieving better pain control, as well as early mobilization and loading for these patients. In the literature, there are also better morbidity and mortality rates in patients who were approached early, leading to shorter hospital stays and fewer postoperative complications.^5–9^ In this study, we present a retrospective evaluation of cases operated on in a hospital service in São Paulo – SP – Brazil between 2020-2024.

## OBJECTIVES

To analyze the outcomes related to partial and total arthroplasties in elderly patients with femoral neck fractures in a hospital service in the city of São Paulo.

1. Analyze perioperative complications (incidents during anesthesia, intraoperative fracture, drop in hemoglobin in the immediate postoperative period, death)
2. Analyze postoperative complications (need for reoperation, infection, need for blood transfusion, deep vein thrombosis – DVT or pulmonary embolism – PE)
3. Analyze time to surgery, need for Intensive Care Unit (ICU), and length of hospital stay.
4. Measure progress in motor physiotherapy (sitting and standing)

## MATERIALS AND METHODS

This is a retrospective observational study in a single hospital center. Approved by the ethics committee, protocol number: 87627325.6.0000.5450.

A review of the medical records of patients who underwent hip arthroplasty between January 1, 2020, and June 30, 2024, was conducted.

### Inclusion criteria

- Age ≥ 65 years;
- Radiographically confirmed femoral neck fracture (Garden I-IV);
- Partial and total hip arthroplasty

### Exclusion criteria

- Pathological fractures (e.g., metastatic);
- Polytrauma patients (to avoid bias from multiple injuries);
- Incomplete follow-up data;
- Elective arthroplasties;
- Patient using anticoagulants (dabigatran, rivaroxaban, apixaban, edoxaban).

### Access to the medical record was through an electronic system. In addition to demographic information, the surgical information collected includes

- Operative time (minutes)
- Surgical access route
- Serum hemoglobin variation (ΔHb)
- Presence of intraoperative fracture
- Need for ICU and length of stay
- Outcomes at 7 days, 30 days, and 6 months
- Time to progress in motor physiotherapy to sitting and standing
- Complications: need for reoperation, infection, blood transfusion; venous thrombosis; death.

### Data Analysis

For statistical analysis, categorical variables were described in absolute and relative frequencies, being compared between groups using Fisher's exact test or chi-square test, as applicable. Continuous variables were described as mean and standard deviation or median and interquartile range, depending on the data distribution, and compared using Mann-Whitney tests. The significance level adopted was 5% (p < 0.05). The analyses were conducted using Jamovi software v2.3.

## RESULTS

The sample consisted of 198 patients with femoral neck fractures, with an average age of 84 years (SD = 7). There was a predominance of females, representing 80.2% of the sample (n = 158), and males 19.8% (n = 39).

The time to surgery had an average of 2.3 days (SD = 2.1). Of the total, 71.6% of the patients (n = 141) underwent surgery within two days after admission (early surgery), and 28.4% (n = 56) were operated on after this period (late surgery).

In the comparison between the groups, the average age was slightly lower in the early group (M = 83.85 years; SD = 7.79) compared to the late group (M = 85.66 years; SD = 6.79), with no statistically significant difference (p = 0.191). The distribution by sex was also similar between the patients operated on early or late, with a predominance of women in both (early group: 82.3% female; late group: 75% female; p = 0.248).

The operative time in the total sample was, on average, 99.1 minutes (SD = 37.5). Among the patients operated on early, the average was 110.6 minutes (SD = 45.5), while among those operated on late, the average was 87.5 minutes (SD = 23.5), with no statistically significant difference between the groups (p = 0.270). (Table 1)

**Table 1:** Operative time in patients undergoing surgery.

	Total				Early				Late				
	N	Med	Min	Max	N	Med	Min	Max	N	Med	Min	Max	p
Operative Time (minutes)	32	90	55	210	16	105	55	210	16	90	60	135	0.191
												

Note: N= Frequency; Med= Mean; Min= Minimum; Max= Maximum; p = p-value.

The average variation of hemoglobin was 2.3 g/dL (SD = 1.4) in the total sample, being 2.4 g/dL (SD = 1.4) in the early group and 2.3 g/dL (SD = 1.5) in the late group, also with no significant difference between the groups (p = 0.872). Regarding the type of arthroplasty, most patients underwent partial arthroplasty (62.9%, N = 124), with a higher frequency in the late group (71.4%, N = 40) compared to the early group (59.6%, N = 84), although without statistically significant difference (p = 0.142). (Table 2)

**Table 2:** Type of arthroplasty performed.

		Total	Early	Late	
		N	N	N	p
Type of arthroplasty	Partial	124	84	40	0.142
	Total	73	57	16	

Note: N= Frequency; p = p-value.

### Outcome in the immediate postoperative period

**Figure 1:**
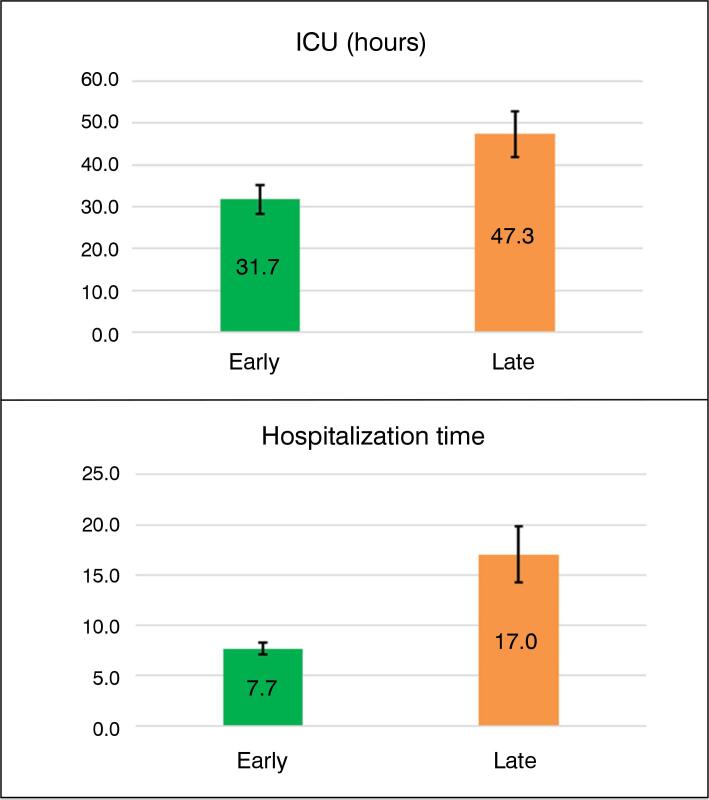
Length of stay in the ICU and hospitalization time of patients undergoing surgery.

Note: Mean ± Standard Error.

In relation to the immediate postoperative variables, it was observed that the length of stay in the ICU was significantly longer (p = 0.002) in the late surgery group (M = 47, SD = 40) compared to the early surgery group (M = 31, SD = 41), with a small to moderate effect size (ES = 0.292). Similarly, the total length of hospitalization was considerably longer in the Late Surgery group (M = 17 days) compared to the Early group (M = 7 days), with statistical significance (p = 0.001) and a moderate-high effect size (ES = 0.695). (Table 3 and Figure 1).

**Table 3:** Postoperative variables in patients undergoing surgery.

	Total		Early		Late		
	N	Med	N	Med	N	Med	p
ICU (hours)	194	25	139	24	55	36	0.002
Length of stay (Days)	197	7	141	6	56	11	0.001
Time to sitting (days)	185	1	134	1	51	1	0.479
Time to standing (days)	162	2	118	2	44	3	0.106

Note: N= Frequency; Med= Mean; p = p-value.

Regarding the time to progress to sitting, the Late group (M = 2.3; SD = 2.8) showed a slightly higher average compared to the early surgery group (M = 1.7; SD = 1.1), with no statistically significant difference between them (p = 0.479). The time to standing also showed no significant difference between the groups (p = 0.106).

Almost all patients were admitted to the ICU, with only two patients from the Early group not requiring intensive care. The occurrence of sitting was high in both groups (92.7% in the Early group and 89.8% in the Late group), with no significant difference (p = 0.547). Similarly, standing was achieved by 84.4% of patients in the Early group and by 77.1% in the Late group, also with no statistically significant difference (p = 0.284). These findings indicate that, despite differences in the length of hospitalization and stay in the ICU, immediate functional progression was similar between the groups, as shown in Table 4.

**Table 4:** Patients who required ICU after surgery and those who achieved sitting or standing.

		Total	Early	Late	
		N	N	N	p
ICU	No	2	2	0	1.000
	Yes	192	137	55	
Sitting	No	12	7	5	0.547
	Yes	133	89	44	
Standing	No	26	15	11	0.284
	Yes	118	81	37	

Note: N= Frequency; p = p-value.

The only complications or outcomes observed in the 7-day postoperative period were deep vein thrombosis (DVT), prosthesis dislocation, and pulmonary embolism (PE), all occurring exclusively in the group undergoing early surgery, while readmissions were recorded only in the group undergoing late surgery. A total of 4 deaths (2.84%) were recorded, with 3 (3.23%) in the early group and 1 (2.08%) in the late group, with no statistically significant difference between the groups (p ≈ 1). (Table 5)

**Table 5:** Possible complications and deaths in patients undergoing surgery.

		Total	Early	Late	
		N	N	N	p
Infection (30 days)*	No	191	138	53	1
	Yes	6	3	3	
Infection (180 days)*	No	194	139	55	1
	Yes	3	2	1	
DVT (7 days)*	No	196	140	56	1
	Yes	1	1	0	
DVT (30 days)*	No	196	141	55	0.284
	Yes	1	0	1	
Death (7 days)	No	137	90	47	1
	Yes	4	3	1	
Death (30 days)	No	72	42	30	0.311
	Yes	4	1	3	
Death (180 days)	No	61	36	25	0.688
	Yes	6	3	3	
Complications	No	176	129	47	0.121
	Yes	21	12	9	

Note: N= Frequency; p = p-value.

In the 30 days following surgery, infections were recorded in 6 patients (3.05%), with 3 in the early surgery group (6.38%) and 3 in the late surgery group (8.82%). Only one case of DVT was identified (1.37%), belonging to the early group (3.23%). There were two readmissions in total, one in each group, with the late group readmission occurring due to worsening clinical conditions. Additionally, four new deaths were recorded during this period, with one in the early group (2.33%) and three in the late group (9.09%), totaling eight deaths in the first month postoperatively.

In the follow-up up to six months after surgery, three cases of infection were identified (4.17%), of which two occurred in the early surgery group (4.88%) and one in the late surgery group (3.23%). Six deaths were recorded during this period, evenly distributed between the groups (n = 3 each), totaling 14 deaths by the sixth month of postoperative follow-up, with seven in each group.

In total, 21 patients (10.7%) experienced some type of complication after surgery, with 18 (9.1%) corresponding to non-fatal complications. In the group undergoing early surgery, 12 patients (8.5%) experienced complications, while in the late group this number was 9 patients (16.1%). Despite the apparent difference between the groups, the comparison of proportions did not reveal statistical significance (p = 0.121).

Throughout the six months of postoperative follow-up, complications occurred in both groups, with no statistical difference.

## DISCUSSION

Femoral neck fractures in the elderly represent a significant public health challenge, and surgical management, especially arthroplasty, is an already standardized approach. The discussion regarding the timing of surgery and its impacts on outcomes such as length of stay, complications, and mortality is central in the literature. This retrospective study aimed to analyze the impact of timing for surgery (early vs. late) in elderly patients undergoing arthroplasty for femoral neck fracture.

Our results did not demonstrate statistically significant differences in clinical and functional outcomes between elderly patients with femoral neck fractures undergoing early surgery compared to those undergoing late surgery. Although the group undergoing late surgery had a longer stay in the ICU and hospital, with the ICU showing a significant difference, the immediate functional outcomes (sitting and standing) and the rate of postoperative complications, including mortality, were similar between the groups over the six months of follow-up.

In total, 10.7% of patients experienced complications, with 9.1% being non-fatal complications. The distribution of adverse events was numerically higher in the late group (16.1% vs. 8.5%), but without statistical significance (p = 0.121). These findings indicate that, although early surgery is associated with shorter hospitalization and ICU stay, it did not result in a significant reduction in complications or mortality when compared to late surgery. Thus, the data suggest that the time to surgery was not a determining factor for the main clinical outcomes analyzed. However, the finding of shorter ICU time is consistent with the literature. Schneider et al.^10^, in a retrospective review, identified that prolonged hospitalization after arthroplasty for hip fractures in the elderly is associated with an increased risk of mortality at 30 days. Although our study did not directly associate prolonged hospitalization with a statistically significant increased risk of mortality, the correlation between longer hospitalization and late surgery in our cohort reinforces the importance of optimizing care flow to reduce hospital stay. The study by Thornburgh et al.,^11^ although focused on rehabilitation in a private setting, also recognizes that hospitalization time is a relevant outcome influenced by various factors after hip fracture.

Regarding postoperative complications and mortality, we found no statistically significant differences between the early and late surgery groups, despite a numerical trend of more complications in the late group. This finding is particularly relevant when compared to Ko et al.,^12^ who investigated trends in hospitalization time, complication rates, and mortality in patients with hip fractures. The authors observed a substantial decrease in hospitalization time but concluded that the complication and mortality rates remained comparable across the different periods analyzed. This suggests that while early surgery may reduce hospitalization time, other factors may play a more determining role in the occurrence of complications and survival.

The presence of certain short-term complications exclusively in the early surgery group in our study, while early readmissions were seen only in the late group, raises questions about the nature of the complications associated with each surgical timing strategy. It could be that patients undergoing late surgery were, on average, more clinically stable before the intervention, or that the wait allowed for the optimization of conditions that reduced acute risks but increased length of stay. Thornburgh et al.^11^ identified a series of factors, such as comorbidities, delirium, and home support, that influence hospitalization time and discharge destination, which may modulate risk profiles at different surgical times.

Our results indicate that, although early surgery is associated with a shorter hospitalization time and need for ICU, its impact on reducing complications or mortality has not been statistically proven. This shows that comprehensive clinical management of elderly patients with hip fractures is essential to optimize outcomes, as suggested by Ko et al.^12^ and Schneider et al.^10^.

### Limitations

This study is not without limitations. We believe that larger samples may be of great value for a statistic with greater comparative power and more conclusive results.

Our sample also has a quite high average age. This becomes a bias as it is very common for very elderly patients to be referred to the ICU in the immediate postoperative period as a precaution, leading to an increase in the number of ICU days, even in patients who would eventually tolerate recovery directly in the ward, for example.

Finally, our sample is also unbalanced, with a predominance of the female sex. We consider this finding related to the higher prevalence of osteoporosis in the female population, associated with a prolonged menopause duration.

## CONCLUSION

We found in this study that late surgery in elderly patients with femoral neck fractures is associated with a significant increase in the length of stay in the ICU and the total hospital stay, with the latter data showing a moderate to high effect size. No statistically significant differences were observed in the rates of overall postoperative complications or mortality at 7, 30, and 180 days between the groups undergoing early or late surgery. Immediate functional progression (sitting and standing) also showed no significant differences between the groups. Although early surgery may contribute to reducing the length of hospital stay, the complexity of managing hip fractures in the elderly requires a multifaceted approach.

## Data Availability

The authors confirm that all data supporting the findings of this study are available within the article.

## References

[1] Gullberg B, Johnell O, Kanis JA (1997). World-wide projections for hip fracture. Osteoporos Int.

[2] Ribeiro MCF, Ribeiro MEF, Santos G da S dos, Lima AK de S, Leonel BMC, Trindade MMM de C (2024). Perfil epidemiológico das internações por fratura de fêmur no Brasil entre 2019 a 2023. Braz. J. Implantol Health Sci.

[3] Soares DS, de Melo LM, da Silva AS, Martinez EZ, Nunes AA (2014). Fraturas de fêmur em idosos no Brasil: análise espaço-temporal de 2008 a 2012. Cad Saúde Pública.

[4] Garden RS (1961). LOW-ANGLE FIXATION IN FRACTURES OF THE FEMORAL NECK. Bone Joint J.

[5] Foex BA, Russell A (2018). BET 2: CT versus MRI for occult hip fractures. Emerg Med J.

[6] Queiroz RD, Borger RA, Heitzmann LG, Fingerhut DJP, Saito LH (2022). Intracapsular Femoral Neck Fractures in the Elderly. Rev Bras Ortop (Sao Paulo).

[7] Taylor BC, Schreiner PJ, Stone KL, Fink HA, Cummings SR, Nevitt MC (2004). Long-term prediction of incident hip fracture risk in elderly white women: study of osteoporotic fractures. J Am Geriatr Soc.

[8] Foss NB, Kehlet H (2006). Hidden blood loss after surgery for hip fracture. J Bone Joint Surg Br.

[9] Dyer SM, Perracini MR, Smith T, Fairhall NJ, Cameron ID, Sherrington C, Falaschi P, Marsh D (2021). Orthogeriatrics: The Management of Older Patients with Fragility Fractures [Internet].

[10] Schneider AM, Mucharraz C, Denyer S, Brown NM (2022). Prolonged hospital stay after arthroplasty for geriatric femoral neck fractures is associated with increased early mortality risk after discharge. J Clin Orthop Trauma.

[11] Thornburgh Z, Samuel D (2022). Factors Influencing Length of Stay and Discharge Destination of Patients with Hip Fracture Rehabilitating in a Private Care Setting. Geriatrics (Basel).

[12] Ko YS, Kang SY, Lee HJ, Kim HS, Yoo JJ (2024). Trends in Hospital Stay, Complication Rate, and Mortality in Hip Fracture Patients: A Two-Decade Comparison at a National Tertiary Referral Center. J Clin Med.

